# Endoscopic management is the preferred treatment modality for a Grade III vesicoureteric reflux with breakthrough infections in a young girl

**DOI:** 10.4103/0970-1591.44251

**Published:** 2008

**Authors:** S. S. Joshi

**Affiliations:** Department of Urology, Jalsok Hospital, Mumbai 400 026

**Keywords:** VUR, deflux injection, open surgical techniques

## Abstract

Endoscopic injection treatment for VUR appears to have less success rate than open surgical treatment, even in Gr 3 VUR. Economics of use of deflux injection and its success rate do not suit Indian milieu. To achieve high success rate of Atlanta group in endoscopic injection treatmnent , requires a longer learning curve and yet it has not achieved success rate of 96-98% achieved by open surgical techniques. Recent addition of modified extravesical reimplantation technique has reduced significantly the post operative pain and patient can be discharged within 2 days from the hospital.

Indications for surgical management for Grade III vesicoureteric reflux (VUR) are similar to that for endoscopic injection treatment.

Then why is open surgical treatment better suited than endoscopic management?

The main points to be considered before answering this question are:

- Results of endoscopic treatment are inferior to open surgery.
- Anatomic problems locally like paraureteric diverticulum, complete duplex system have poorer results
- Neurogenic bladders with poor compliance or with detrussor fibrosis have even poorer results with endoscopic management.
- In India, the economics of using Defulx, presently the preferred agent for endoscopic treatment is not suitable for all.

## RESULTS OF INJECTION THERAPY

Elders *et al.*,[1] in their meta-analysis showed that endoscopic therapy results after first injection for Grade 1/2 was 79%, Grade III was 72%, and 65% for Grade 4/5. Following the second injection results improved to 85% overall. Kirsch *et al.*,[2] modified endoscopic injection treatment with hydrodistension and injected intraureterally. Results improved further to 90%. But these have not been validated nor any other unit been able to get these results. American Urology Association guidelines report surgical correction success rate of 96% overall. Contemporary studies by Lavine,[3] Barrieras[4] confirm this high success rate. In fact they even question the necessity of a postoperative cystogram to confirm resolution of VUR.

An Atlanta group led by Dr. Kirsch believe one of the reasons for failure in Grade III or higher grades, is too low volume of Deflux injected and ureteral mucosa fails to coapt. For Grade III or higher grades of VUR require minimum of 1.2-1.6 ml of Deflux. Deflux is available only in 1 cc vial, and costs about Rs.22,000/-. So for adequate injection one will require two vials costing Rs.44,000/-, this is besides other incidental costs of admission for one day, surgeon's fees, hospital charges etc. According to the manufacturers of Deflux, volume of 1 cc was arrived based on their study of the viscocity of Deflux, pressure required to inject without extravasation of Deflux and other factors. The main agent in India for Deflux is in New Delhi but has subagents in most of the metro cities of India. The injections are sent by courier to any part of India on order. We have to pay in full, on order before dispatch from New Delhi for the deflux injection. There is no scheme by which payment is done only if deflux injection is used? Since the success rate is around 72% for Grade III, failures will require one more injection and admission to hospital, to improve ureteral mucosal coaptation!

Dr. Routh *et al.*,[5] on univariate analysis found gender (boys do poorly as compared to girls), age, VUR grade, injection technique (STING or HIT or double HIT) and SURGEON were significant. However, on multivariate analysis, only VUR grade and SURGEON remained significant factors. Surgeon's experience, it appears, is important as shown by Lavell and Skoog[6] *et al.*, who found that the number of times that urothelium was punctured during injection influenced long-term resolution. But how does a surgeon gain experience or shorten the learning curve?

Two local anatomical abnormalities viz. paraureteric diverticulum and complete duplex system also show poor results with injection therapy. Papastoris found the distance between two ureteric orifices important parameters for success. If the distance was <1 cm, resolution of was seen 64.2% and if the distance was >1 cm resolution was seen 77.1%. These results are way behind 100% success of uretero-uretero-ureterostomy or pyelo-ureterostomy!

In short, the economics of Deflux injection does not suit us, neither does its failure rate!

One of the advantages of endscopic injection therapy is its one day admission for the child. Palmer reported on 50 children with bilateral extravesical ureteric reimplant. Patients were discharged on the first postoperative day with oral analgesics. None had urinary retention and success was 100%. Wicher[7] *et al.*. treated 80 patients with unilateral VUR, 20 of whom were treated as inpatient and 60 as outpatient/daycare surgery. Both the groups had extravesical reimplant. Inpatient stay was 31.25 h and outpatient stay was 6.6 h. There was no urinary retention. That there is no urinary retention by extravesical reimplantation was also shown by Riedl. He did extravesical reimplant bilaterally in 85 children and unilaterally in 177 with Grade III/IV VUR predominating. After 5, 10, 15 years follow-up, resolution of VUR was 95-100%. Persistent post-void residue (PVR) was seen in 2% children in unilateral and in 4% in bilateral cases. There were no symptoms and no treatment was offered other than conservative treatment.

The reason for this success of extravesical reimplant, is the modification of surgical technique. Presently, in extravesical reimplant, the ureteral dissection is limited distal to the obliterated umbilical artery, and adequate detrussororraphy. With this technique post-operative retention is not in unilateral reimplantation and is not common even in bilateral reimplantation.

Another great advantage of extravesical reimplant of ureters is that natural straight line from ureteric orifice to kidney is maintained. It is also maintained with the Politano-Leadbetter technique, but this technique does carry a small complication rate. However, this cannot be said of the Cohen technique. But it is important to remember that all the above techniques will give you 96-98% success rate. Hospital stay is two days longer, maybe, but is compensated by the excellent long-term results. Pain is a factor in intravesical procedures, but can be effectively managed with caudal sensorcaine injection and oral analgesics. Children have a remarkable ability to recover from any surgical procedure.

## CONCLUSION

Endoscopic injection therapy (Deflux) is inferior to surgical reimplantation in long-term results, even for Grade III VUR. The economics of Deflux injection also appears not to suit our milieu. There is longer learning curve for a surgeon to approach >72% results as compared to shorter apprenticeship in open surgical procedures.
